# Aging and Audio-Visual and Multi-Cue Integration in Motion

**DOI:** 10.3389/fpsyg.2013.00267

**Published:** 2013-05-23

**Authors:** Eugenie Roudaia, Allison B. Sekuler, Patrick J. Bennett, Robert Sekuler

**Affiliations:** ^1^Department of Psychology, Neuroscience, and Behaviour, McMaster UniversityHamilton, ON, Canada; ^2^Institute of Neuroscience, Trinity College DublinDublin, Ireland; ^3^Volen Center for Complex Systems, Brandeis UniversityWaltham, MA, USA

**Keywords:** ageing, aging, multisensory integration, motion perception, audio-visual integration, multiple cues combination, bounce-stream

## Abstract

The perception of naturalistic events relies on the ability to integrate information from multiple sensory systems, an ability that may change with healthy aging. When two objects move toward and then past one another, their trajectories are perceptually ambiguous: the objects may seem to stream past one another or bounce off one another. Previous research showed that auditory or visual events that occur at the time of disks’ coincidence could bias the percept toward bouncing or streaming. We exploited this malleable percept to assay age-related changes in the integration of multiple inter- and intra-modal cues. The disks’ relative luminances were manipulated to produce stimuli strongly favoring either bouncing or streaming, or to produce ambiguous motion (equal luminances). A sharp sound coincident with the disks’ overlap increased both groups’ perception of bouncing, but did so significantly less for older subjects. An occluder’s impact on motion perception varied with its duration: a long duration occluder promoted streaming in both groups; a brief occluder promoted bouncing in younger subjects, but not older ones. Control experiments demonstrated that the observed differences between younger and older subjects resulted from neither age-related changes in retinal illuminance nor age-related changes in hearing, pointing to weakened inter- and intra-modal integration with aging. These changes could contribute to previously demonstrated age-related perceptual and memory deficits.

## Introduction

How the brain manages to coordinate and integrate information received from multiple sources is one of cognitive neuroscience’s central questions. After all, integration of information from multiple sources is crucial for perception and for other cognitive functions. Many studies have focused on one particular form of integration that holds especial importance: the integration of information from multiple senses (e.g., Soto-Faraco et al., 2003; Yuval-Greenberg and Deouell, 2007; Bruns and Getzmann, 2008; Schutz and Kubovy, 2009; Bizley et al., 2012; Naci et al., 2012). Given the fact that we inhabit a world in which events tend to be multisensory, researchers’ emphasis on multisensory integration is well placed. As is the case with many other cognitive functions, multisensory integration seems to change with age (for review, see Mozolic et al., 2012). In particular, older subjects show greater multisensory enhancement than younger subjects when processing complex audio-visual stimuli, such as speech (Cienkowski and Carney, 2002; Maguinness et al., 2011; Winneke and Phillips, 2011), or when detecting or discriminating simple multisensory stimuli (Laurienti et al., 2006; Peiffer et al., 2007; Mahoney et al., 2011, c.f., Stephen et al., 2010). However, the reasons for enhanced multisensory integration in aging are not well understood. Because various aspects of motion perception are also affected by age (Habak and Faubert, 2000; Norman et al., 2003; Bennett et al., 2007; Andersen and Ni, 2008; Billino et al., 2008; Pilz et al., 2010; Roudaia et al., 2010), we decided to use motion perception as an arena within which to examine age-related changes in multisensory integration. For this purpose, we focused on a visual stimulus whose alternative, competing percepts are strongly influenced by accompanying sound.

This bistable, but malleable, percept arises when an observer observes two identical objects that move steadily toward one another, coincide, and then move apart. The appearance of the objects’ trajectory fluctuates: the two moving objects sometimes appear to stream directly through one another, but sometimes they seem to bounce off one another (Metzger, 1934). The ambiguity of the stimulus is curtailed when a sharp sound is presented as the objects coincide (Sekuler et al., 1997; Shimojo et al., 2001; Watanabe and Shimojo, 2001; Remijn et al., 2004; Sanabria et al., 2004; Zhou et al., 2007). The sound strongly biases the percept, causing the objects to seem to bounce off one another.

The perceptual outcome of the ambiguous visual stimulus also can be biased by changes in the visual display itself. For example, when an opaque occluder obscures the region of the display in which the objects will coincide, the presence of the occluder promotes the appearance that the moving objects streamed through one another (Sekuler and Sekuler, 1999). This result may be related to other conditions in which an object’s perceptual continuity is preserved in the face of a temporary occlusion (Feldman and Tremoulet, 2006). The ability to track an occluded object has obvious potential evolutionary value. In the laboratory, this ability has been studied most intensively with multiple object tracking (Scholl and Pylyshyn, 1999; Alvarez et al., 2005; Horowitz et al., 2006), which has been shown to decline with aging (Trick et al., 2005; Sekuler et al., 2008; Kennedy et al., 2009). Assad and Maunsell (1995) described a class of neurons that may contribute to this preservation of identity by signaling the presence of a briefly occluded moving object.

To preview, we exploited the ambiguous bouncing-streaming percept as a vehicle for assaying possible age-related changes in the integration of both inter- and intra-modal cues for visual motion. Because an occluder’s perceptual impact seems to vary with its temporal properties (Sekuler and Palmer, 1992; Murray et al., 2001; Guttman et al., 2003; Remijn et al., 2004), we simultaneously examined how age affected occlusion’s impact on the bistable, bouncing-streaming percept.

## Experiment One

### Methods

#### Apparatus

The experiment was programed in the Matlab environment (version 7.2) using the Psychophysics and Video Toolboxes, v. 3.0.8) (Brainard, 1997; Pelli, 1997) on a Macintosh G5 computer running OS X (10.4.11). Visual stimuli were presented in a dark room on a 21-inch Sony Trinitron monitor with 1280 × 1024 resolution (pixel size = 0.03°) and a refresh rate of 60 Hz. The display area subtended 38.4 × 30.7° visual angle, at a viewing distance of 57 cm. The mean luminance of the display was 37.5 cd/m^2^, and provided the only light source in the testing room. Head position and viewing distance were stabilized using a forehead/chin support. Auditory stimuli were presented through Harman/Kardan SoundSticks III speakers located at display height, 33° to the left and right of the subject’s mid-sagittal plane. The speakers’ frequency response was 44–20 kHz, ±10 dB. As the speakers’ subwoofer emits a distinct glow when the speakers are powered up, we covered them with black fabric to render them invisible during testing. Sound measurements were made with a Brüel & Kjaer 2239 sound level meter located where a subject’s head would be during the experiment. Subjects’ responses were collected with a standard English language keyboard.

#### Stimuli and design

Two disks (radius = 1.5°) appeared at ±9° horizontal eccentricity and 3° above fixation, and moved horizontally toward each other, overlapped in the middle of the display, and continued on, finally reaching the opposite sides of the display. The disks maintained a constant speed of 12°/s for 1.5 s, after which time they disappeared.

The relative luminances of the two disks were manipulated in order to promote ambiguity of motion, or to strongly favor a particular percept, either bouncing or streaming. In all *Ambiguous* conditions, the two disks had the same luminance with −0.7 contrast with the background. To generate stimuli for two *Unambiguous* conditions, we exploited a previous demonstration that two objects’ perceived trajectories depend upon the way that the objects’ features vary or do not vary over time (Feldman and Tremoulet, 2006). Specifically, in *Unambiguous* conditions, the two disks had −0.85 and −0.55 contrast with the background respectively, and their motion was either unambiguously *Streaming*, so that each disk crossed from one side to the other, or unambiguously *Bouncing*, so that each disk traveled to the point of coincidence and returned to its starting point (see Figure 2).

**Figure 1:**
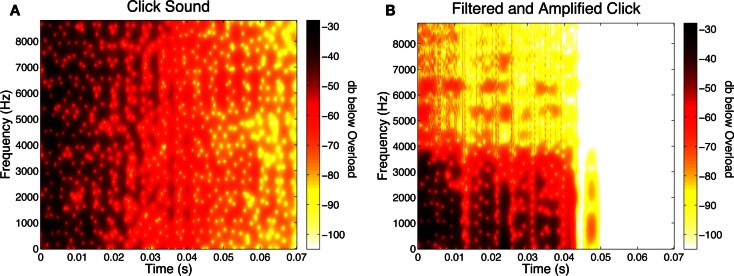
**(A)** Spectrogram of the click sound used in Experiment One. This plot shows the amplitude of different frequencies of the sound over time. The color bar represent values of dBov (decibels below overload, which is the maximum amplitude before signal clipping would occur). In the bar in the spectrogram, darker colors represent higher amplitudes. Note that in the spectrogram stimulus amplitude initially is high at frequencies over the entire spectrum, but gradually diminishes over time. (**B)** Spectrogram of a click used in Experiment Three. The spectrogram shown is for the age-sensitive, filtered click that has been amplified to match the loudness of the unfiltered click. Note that early on, high amplitude is seen only at the lowest frequencies. Notice also, that above ∼3 kHz, amplitudes are less than at the corresponding frequencies in the unfiltered click.

In some conditions, a synthesized click (90 dBC; 0.070 s duration) sounded as the disks were coinciding. The click was produced by modifying the Karplus-Strong algorithm (Karplus and Strong, 1983) to generate a broadband stimulus with sharp onset and flat frequency spectrum. Figure 1A shows this click’s spectrogram^1^. In some conditions, an opaque rectangular occluder (3.14° × 5.4°; contrast = −0.4) was presented above the fixation point and obscured the disks’ coincidence. The duration of the occluder was either *Short* (0.117 s in duration), *Medium* (0.233 s in duration) or *Long* (extending over the entire trial, that is, 1.5 s), centered around the time of coincidence (i.e., 0.75 s).

**Figure 2:**
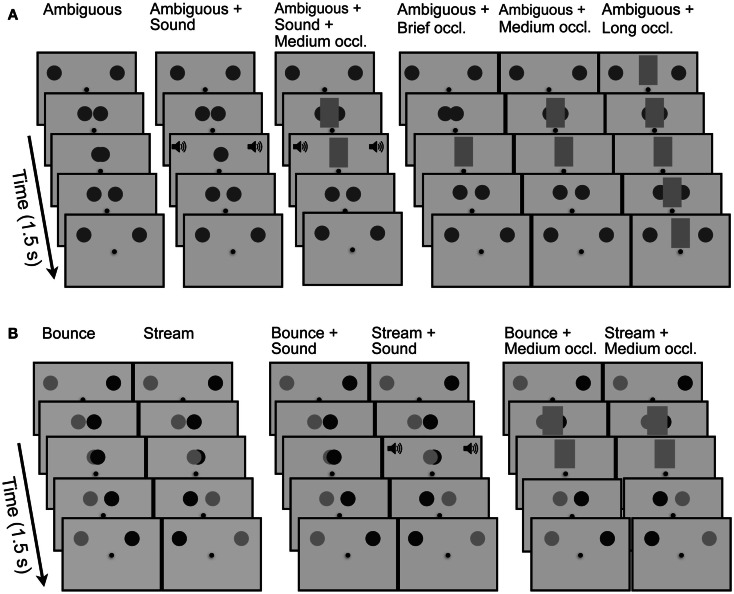
**Schematic representations of the five key events comprising conditions in Experiment One**. **(A)** Key events in the six conditions that are meant to generate perceptually ambiguous motion, that is, motion in which the two equal contrasts disks could be seen either as streaming through one another or bouncing off one another. *Ambiguous*: key events in the basic, control condition; *Ambiguous* + *Sound*: a click sound is presented as the two disks coincide; *Ambiguous* + *Sound* + *Medium occluder*: in addition to the click sound, the disks’ coincidence is obscured by opaque occluder presented for 0.233 s. In the next three conditions, no sound is presented. *Ambiguous* + *Short occluder*: the disks’ coincidence is obscured by opaque occluder presented for 0.117 s; *Ambiguous* + *Medium occluder* the disks’ coincidence is obscured by opaque occluder presented for 0.233 s; *Ambiguous* + *Long occluder*: the location at which the disks would coincide is obscured by opaque occluder presented for 1.5 s, that is, the entire duration of the moving stimulus. **(B)** Key events in the six conditions that promoted unambiguous motion. Two disks of unequal luminances moved toward each other, coincided, and then either returned to their respective starting locations (promoting a percept of bouncing; shown in sequence 1) or continued to the opposite side of the display (promoting a percept of streaming; shown in sequence 2). In sequences 3 and 4, at the moment of the disks’ coincidence, a click sound was inserted into a stimulus that promoted bouncing (sequence 3) or one that promoted streaming (sequence 4). In the final two sequences, a 0.233-s occluder was inserted into a sequence that promoted bouncing (sequence 5) or one that promoted streaming (sequence 6).

There were 12 stimulus conditions. In six conditions, the disks had unbalanced luminances, with three conditions consistent with a bouncing percept and three conditions consistent with a streaming percept. The unambiguous disk motions were presented alone (*Bounce*, *Stream*), together with a sound at coincidence (*Bounce* + *Sound*, *Stream* + *Sound*), or along with a medium duration occluder (*Bounce* + *Medium*, *Stream* + *Medium*). In the remaining six conditions, the disks had equal luminance, rendering their movement trajectory ambiguous. In these conditions, the disks were presented alone (*Ambiguous*), with a sound at coincidence (*Ambiguous* + *Sound*), with occluders of different duration (*Ambiguous* + *Short*, *Ambiguous* + *Medium*, *Ambiguous* + *Long*), or with a sound and an medium duration occluder (*Ambiguous* + *Sound* + *Medium*). This combination of conditions promoted a relatively equal proportion of “Bouncing” and “Streaming” responses, and balanced the distribution of cues (sound and occluders) across conditions of unambiguous and ambiguous motion. Figure 2 illustrates the sequence of events comprising all 12 conditions.

#### Subjects

Thirteen younger and 16 older subjects, who were naïve as to the purposes of the study, participated in this experiment and were compensated for their time at a rate of $10/h. Near and far Snellen acuities and Pelli-Robson contrast sensitivity were measured while subjects wore their customary prescription. All subjects had Snellen acuity of 20/30 or better, as well as good contrast sensitivity. Older subjects averaged 29.1/30 on the Mini-Mental State Exam (Folstein et al., 1975). To be included in the analyses, subjects had to report “streaming” 70% of the time in the *Stream* condition and report “bouncing” 70% of the time in the *Bounce* condition. This criterion resulted in the exclusion of one younger and three older subjects. The demographic information for the remaining 12 younger subjects and 13 older subjects included in the analyses is presented in Table 1.

**Table 1:** **Mean ± 1 SD age, near and far logMAR acuity, Pelli-Robson contrast sensitivity, and Mini-Mental State Examination (MMSE)**.

Expt	*N* (M:F)	Age (years)	Near acuity (logMAR)	Far acuity (logMAR)	Pelli-Robson (log contrast)	MMSE (max = 30)
1	13 (7:6)	67.7 ± 6.25	0.03 ± 0.11	−0.04 ± 0.09	1.95 ± 0	29.1 ± 0.76
	12 (2:10)	20.0 ± 1.8	−0.13 ± 0.05	−0.09 ± 0.08	1.95 ± 0	
2	8 (4:4)	24.8 ± 2.4	−0.14 ± 0.05	−0.09 ± 0.14	1.95 ± 0	
3	13 (4:9)	20.1 ± 2.8	−0.14 ± 0.05	−0.11 ± 0.13	1.93 ± 0.04	

#### Procedure

The McMaster University Research Ethics Board approved the experimental protocol. Written informed consent was obtained from all subjects prior to their participation in the experiment.

Prior to beginning the experiments, subjects were told that they would be asked to judge whether two disks that traveled toward each other streamed past each other, or bounced off of each other. To demonstrate these two situations, subjects were shown four examples of a red and blue disk that seemed to stream past each other and four examples of a red and blue disk that seemed to bounce off each other. Subjects were then informed that sometimes the two disks will have different luminance, or have the same luminance. Subjects were also informed that a brief sound, an occluder, or both a sound and an occluder will sometimes be presented during the disks’ motion. Subjects were told to ignore these events and to concentrate on reporting the disks’ pattern of motion.

Each trial began with the presentation of a black fixation point (diameter = 0.25°) at the center of the screen. Subjects were instructed to fixate this location throughout each trial. The fixation point flickered at 10 Hz for 0.3 s to attract the subject’s attention. After a delay of 0.1 s, the disks appeared and moved steadily across the screen for 1.5 s. After the disks disappeared, the letters “B” and “S” appeared on either side of fixation and remained on the screen until the subject’s response. The mapping of “B” and “S” to right and left sides was counterbalanced in each group, so that half the subjects in each group responded by pressing “B” with their dominant hand and “S” with their non-dominant hand. No response feedback was given. The following trial began 1.5 s after the response.

Each subject completed nine blocks of trials, each containing four repetitions of each of 12 stimulus conditions presented in randomly intermixed order, resulting in a total of 36 trials per condition. The experimental blocks were preceded by a practice block of 24 trials consisting of two trials per condition in randomly intermixed order.

After the experiment, older subjects also completed a short hearing test to determine whether they could successfully hear our sound stimulus. In the test, 10 sounds were played at random intervals ranging from 3 to 10 s. Subjects were asked to press the space-bar on a computer keyboard as soon as they heard the sound. Every subject successfully detected all 10 sounds. Two subjects each gave one false alarm, that is, they pressed the space-bar when no sound had been presented. One other subject committed eight false alarms. That subject had also shown low accuracy in conditions of unambiguous motion, and was excluded from further data analyses.

### Results

All statistical analyses were performed using the statistical computing environment R (The R Project for Statistical Computing, 2012). The proportion of “Bouncing” and “Streaming” responses was calculated for each condition. As mentioned earlier, for four subjects (one younger), the unambiguous conditions failed to produce their intended effect: one younger subject showed 0.47 “Streaming” judgments in the *Stream* condition, one older subjects produced 0.67 “Bouncing” judgments the *Bounce* condition and only 0.56 “Streaming” judgments in the *Stream* condition, and two other older subjects produced just 0.56 and 0.44 “Bouncing” judgments in the *Bounce* condition. All data from these subjects were excluded.

### Unambiguous conditions results

**Figure 3:**
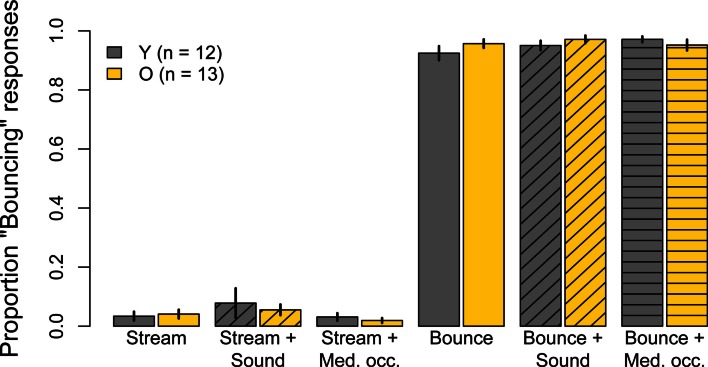
**Experiment One results: mean Pr(“Bouncing”) responses for younger subjects (gray) and older subjects (yellow) for six conditions in which disk luminances had been unbalanced in order to maximize “Streaming” and “Bouncing” judgments**. Data are for 12 younger and 13 older subjects. Error bars are ±1 standard error of the mean.

The proportion of “Bouncing” responses in the six unambiguous conditions whose unbalanced disk luminances were meant to promote perceptual consistency are shown in Figure 3. With the exception of the subjects whose data were excluded, the stimuli in these conditions were equally effective for younger and older subjects. Moreover, for either group, the addition of a sound or of an occluder to these stimuli had no effect on perceptual judgments. The proportion of “Bouncing” responses in both age groups in the unambiguous *Bounce* and unambiguous *Stream* conditions were analysed in two separate 2 (age) × 3 (condition: *alone*, with *Sound*, with *Occluder*) split-plot ANOVAs. The main effects of age, condition, and the Age × Condition interaction were not significant for either the *Bounce* condition (age: *F*(1, 23) = 0.42, *p* = 0.53; condition: *F*(2, 46) = 1.58, $\hat{\epsilon }$ = 0.91, *p* = 0.22; Age × Condition: *F*(2, 46) = 2.04, $\hat{\epsilon }$ = 0.98, *p* = 0.14) or the *Stream* condition (age: *F*(1, 23) = 0.11, *p* = 0.74; condition: *F*(2, 46) = 3.56, $\hat{\epsilon }$ = 0.58, *p* = 0.06; Age × Condition: *F*(2, 46) = 0.45, $\hat{\epsilon }$ = 0.59, *p* = 0.54).

### Ambiguous condition results

**Figure 4:**
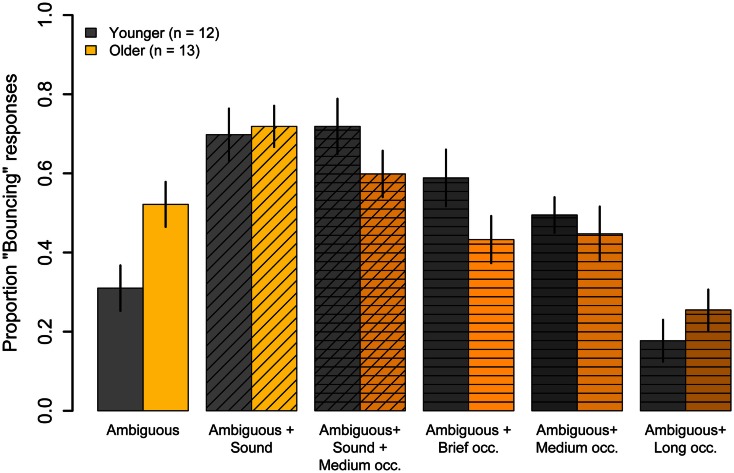
**Experiment One results: mean Pr(“Bouncing”) responses for younger subjects (gray) and older subjects (yellow) in six conditions where the motion of the disks was ambiguous**. The disks were accompanied by a sharp sound at the point of coincidence (diagonal hatching), or an opaque occluder of different durations that obscured the point of coincidence (horizontal hatching), or both a sound and an occluder (horizontal and diagonal hatching). Data are for 12 younger and 13 older subjects. Error bars are ±1 standard error of the mean.

Figure 4 shows results for the six conditions where the disks had equal luminance and, therefore, where motion was perceptually ambiguous. When the disks were presented alone (*Ambiguous* condition), younger subjects showed a bias toward “Streaming” responses (*M* = 31%, *t*(11) = −3.30, *p* = 0.007), whereas older subjects showed no significant bias toward either “Bouncing” or “Streaming” (*M* = 52%, *t*(12) = 0.38, *p* = 0.71). The proportion “Bouncing” responses was significantly lower in younger subjects compared to older subjects (*t*(23) = −2.61, *p* = 0.02).

#### Effect of sound

Presenting a sound at the point of coincidence in the *Ambiguous* condition significantly increased the proportion of “Bouncing” responses given by younger subjects (31 vs 70%, *t*(11) = 6.94, *p* < 0.001) and by older subjects (52 vs 72%, *t*(12) = 5.05, *p* < 0.001). However, the increase for younger subjects was significantly greater than that for older subjects (*F*(1, 23) = 8.05, *p* = 0.009). For both age groups, the *Ambiguous* + *Sound* condition evoked significantly fewer “Bouncing” responses than did the unambiguous *Bounce* condition (younger: *t*(11) = −3.32, *p* = 0.007; older: *t*(11) = −3.83, *p* = 0.002), suggesting that the significant interaction was probably not the result of some ceiling effect for older subjects.

#### Effect of occluder

The presentation of an opaque occluder during the disks’ motion influenced the percept of the disks’ ambiguous motion. However, the magnitude and sign of the effect varied with the duration of the occluder and with age (see bars with horizontal hatching in Figure 4). This observation was confirmed by a 2 (age) × 4 (condition: no occluder, *Short*, *Medium*, and *Long* occluder durations) split-plot ANOVA that revealed a significant main effect of condition (*F*(3, 69) = 112.6, *p* < 0.001, $\hat{\epsilon }$ = 0.85) and a significant Age × Condition interaction (*F*(3, 69) = 4.75, *p* = 0.007, $\hat{\epsilon }$ = 0.85). The main effect of age was not significant (*F*(1, 23) = 0.16, *p* = 0.69).

To decompose the interaction, we examined the effects for different occluder durations separately for each age group. For younger subjects, both the *Brief* (*M* = 59%) and *Medium* (*M* = 49%) duration occluders significantly increased the proportion “Bouncing” responses relative to the *Ambiguous* condition (*Brief:* *t*(11) = −4.17, *p* = 0.001; *Medium*: *t*(11) = −2.84, *p* = 0.02). For older subjects, however, the *Brief* (*M* = 43%) and *Medium* (*M* = 45%) duration occluders had no significant effect on proportion “Bouncing” responses relative to the *Ambiguous* condition (*Brief:* *t*(12) = 1.24, *p* = 0.23; *Medium*: *t*(12) = 1.10, *p* = 0.29). Finally, the sustained occluder promoted “Streaming” responses in younger subjects (*M* = 18%) and in older subjects (*M* = 25%), consistent with previous reports in young adults (Sekuler and Sekuler, 1999). Proportion “Bouncing” with the *Long* duration occluder did not differ significantly from the *Ambiguous* condition in younger subjects (*t*(11) = 1.6, *p* = 0.14), but was significantly lower in older subjects (*t*(12) = 3.18, *p* = 0.008).

#### Effect of combined sound and occluder

Proportion “Bouncing” evoked by the combination of an opaque occluder and a sound click at the point of coincidence of the disks is shown in Figure 4 (cross-hatched bars). This condition promoted “Bouncing” responses in younger (*M* = 72%) and older (*M* = 60%) subjects. In younger subjects, the combination of both transient events significantly increased Pr(“Bouncing”) compared to the *Ambiguous* condition (*t*(11) = 5.47, *p* = 0.0002), but the bouncing bias was not significantly different from that evoked in the *Ambiguous* + *Sound* condition (*M* = 72 vs *M* = 70%, *t*(11) = 0.31, *p* = 0.76), indicating that the effects of the sound and the medium occluder were sub-additive. In older subjects, on the other hand, Pr(“Bouncing”) evoked in the *Ambiguous* + *Sound* + *Medium Occluder* did not differ significantly from Pr(“Bouncing”) seen in the *Ambiguous* condition (*t*(12) = −1.10, *p* = 0.29). Moreover, the addition of a medium occluder significantly reduced the older group’s proportion “Bouncing” responses compared to the *Ambiguous* + *Sound* condition (60 vs 72%, *t*(12) = −2.13, *p* = 0.05).

## Experiment Two: Retinal Illuminance Control

This experiment examined the possibility that differences between younger and older results seen in Experiment One might have resulted from the reduction in retinal illuminance that accompanies aging. As a result of senile miosis, while viewing the background luminance of our display, an average 68-year-old’s pupil diameter would be 4.70 mm, while an average 20-year-old’s pupil diameter would be 6.60 mm (Winn et al., 1994). The accompanying age-related difference in pupil area would reduce the average older subject’s retinal illuminance by ∼2×. Previous research found that changes in display luminance affect the perceived speed of moving objects (Hammett et al., 2007), allowing for the possibility that age-related reductions in retinal illuminance affected the disks’ perceived speed, thereby promoting group differences in the bouncing/streaming percept (Hammett et al., 2007). To check this, we tested a group of younger subjects who viewed the stimulus display through neutral density filters chosen to reduce retinal illuminance considerably in excess of what would have been expected in Experiment One from senile miosis alone.

### Methods

#### Subjects

Twelve younger subjects participated in this experiment and were compensated for their time at a rate of $10/h. None had participated in Experiment One. Four subjects’ data were excluded because their accuracy in the unambiguous *Bounce* condition was <70% (accuracy range: 12–68%, mean: 43%). Demographic information for the eight remaining subjects is presented in Table 1.

In addition, to assess the stability of older subjects’ performance in Experiment One, all older subjects who participated in Experiment One were re-tested in this experiment.

#### Apparatus

The apparatus was the same as in Experiment One. For younger subjects, display luminance was varied by interposing neutral density filters between a subject and the display, reducing display luminance by ≈90%, from 39.5 to 4.24 cd/m^2^. Note that this reduction far exceeds the reduction in retinal illumination that would have resulted from normal age-related reduction in pupil size (senile miosis).

#### Stimuli and procedure

Half of the conditions used in Experiment One were used in this experiment: *Bounce*, *Stream*, *Ambiguous* presented with no accompanying sound, and *Bounce*, *Stream*, and *Ambiguous* each accompanied by the click sound at the moment of the disks’ coincidence. As in Experiment One, there were nine blocks of trials comprising four trials of each condition in random order, which were preceded by a practice block comprising two trials of each condition. Half the younger subjects completed the six conditions first without neutral density filters and then again with neutral density filters, whereas the other half of the subjects followed the opposite order. Older subjects completed the six conditions once, always without neutral density filters.

### Results

#### Effect of neutral density filters

**Figure 5:**
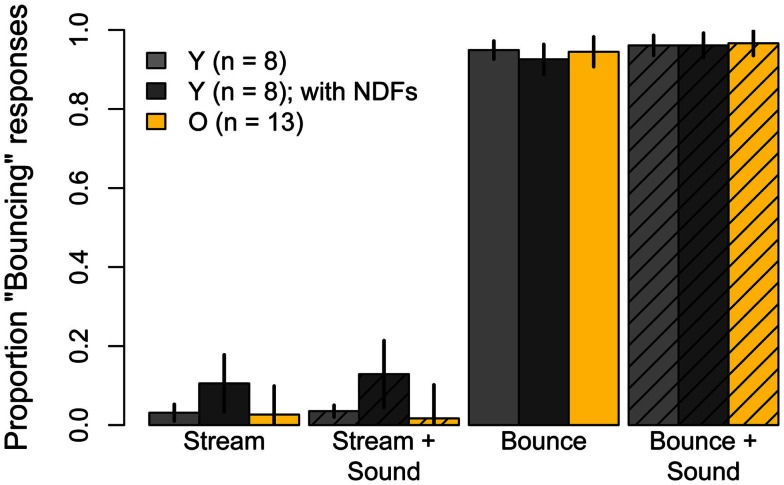
**Mean Pr(“Bouncing”) responses produced in the unambiguous conditions of Experiment One: Bouncing and Streaming motion and Bouncing and Streaming motion accompanied by the sound used in Experiment One**. Light gray bars represent data for eight younger subjects when they viewed the stimuli normally, without neutral density filters, and the dark gray bars represent results obtained when subjects viewed the stimuli through three neutral density filters. Yellow bars represent data of 13 older subjects who participated in Experiment One and were tested again in these conditions without neutral density filters. Error bars are ±1 standard error of the mean.

Figure 5 shows results for the *Bounce* and *Stream* conditions, with and without sound, for younger subjects viewing the stimuli without neutral density filters (light gray bars) and with neutral density filters (dark gray bars). The effect of luminance on proportion of bouncing responses in the unambiguous conditions was analyzed with two separate 2 (sound) × 2 (luminance) repeated-measures ANOVAs. For bouncing conditions, the main effects of sound, luminance, and the Sound × Luminance interaction were not significant (sound: *F*(1, 7) = 1.13, *p* = 0.32; luminance: *F*(1, 7) = 0.14, *p* = 0.71; Luminance × Sound: *F*(1, 7) = 0.51, *p* = 0.50). For streaming conditions, the main effect of sound was significant (*F*(1, 7) = 8.79, *p* = 0.02), as the presentation of the sound increased proportion “Bouncing.” The main effect of luminance was not significant (*F*(1, 7) = 1.46, *p* = 0.27) and the Luminance × Sound interaction was not significant (*F*(1, 7) = 0.69, *p* = 0.43).

**Figure 6:**
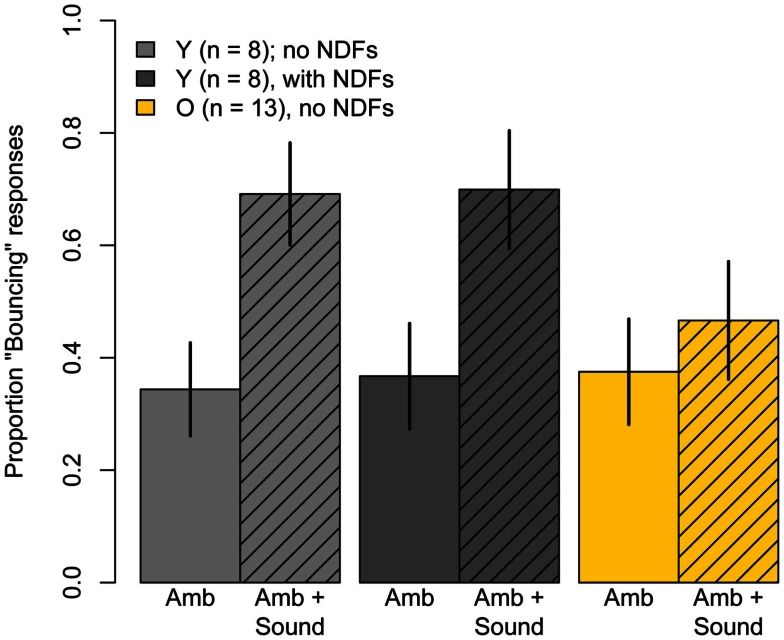
**Mean Pr(“Bouncing”) responses produced in the ambiguous conditions of Experiment Two: *Ambiguous* motion and *Ambiguous* motion accompanied by the sound used in Experiment One**. Light gray bars represent data for eight younger subjects when they viewed the stimuli normally, without neutral density filters, and the dark gray bars represent results obtained when subjects viewed the stimuli through three neutral density filters. Yellow bars represent data of 13 older subjects who participated in Experiment One and were retested in these conditions without neutral density filters. Error bars are ±1 standard error of the mean.

Figure 6 shows results for the *Ambiguous* and *Ambiguous* + *Sound* conditions viewed with and without neutral density filters. A 2 (sound) × (luminance) repeated-measures ANOVA revealed a significant main effect of sound (*F*(1, 7) = 13.9, *p* = 0.007), no significant main effect of luminance (*F*(1, 7) = 0.04, *p* = 0.85), and no significant Sound × Luminance interaction (*F*(1, 7) = 0.04, *p* = 0.86). Thus, the ≈9.5× reduction in luminance had no discernible effect on the proportion of “Bouncing” responses evoked in the *Ambiguous* condition, or on the increase in proportion “Bouncing” by the brief sound click.

#### Replication of older subjects’ performance?

Figures 5 and 6 also show results for 13 older subjects who participated in Experiment One and were re-tested with six conditions in this experiment. To examine the test-retest reliability of older subjects’ performance, proportion “Bouncing” for the six conditions used in this experiment were compared with performance in Experiment One using a 6 (condition) × 2 (time) repeated-measured ANOVA. The main effects of time, condition, and the Condition × Time interaction were all significant (time: *F*(1,12) = 10.3, *p* = 0.007; condition: *F*(5,60) = 217.7, $\hat{\epsilon }$ = 0.37, *p* < 0.001; Condition × Time: *F*(5,60) = 6.47, $\hat{\epsilon }$ = 0.28, *p* = 0.01). To decompose the interaction, we evaluated the simple main effect of time for each condition separately. The simple main effect of time was significant in the *Ambiguous* + *Sound* condition (*F*(1, 12) = 12.7, *p* = 0.004) and in the *Stream* + *Sound* condition (*F*(1, 12) = 8.98, *p* = 0.01), with lower Pr(“Bouncing”) in Experiment Two in both cases. Proportion of “Bouncing” responses for the *Ambiguous* condition was also lower in Experiment Two, but this difference was not statistically significant (*F*(1, 12) = 4.02, *p* = 0.07) and, as in Experiment One, average proportion “Bouncing” was not significantly different from 50% (*t*(12) = −1.87, *p* = 0.09). Proportion “Bouncing” in all other conditions did not differ between experiments (*F*(1, 12) < 1.06, *p* < 0.32).

Unlike in Experiment One, Pr(“Bouncing”) in the *Ambiguous* condition did not differ significantly different between age groups (*t*(19) = −0.29, *p* = 0.77). However, similar to Experiment One, the increase in Pr(“Bouncing”) associated with the presentation of the sound click was smaller in older subjects compared to younger subjects (*F*(1, 19) = 6.14, *p* = 0.02). Indeed, the effect of sound on proportion “Bouncing” responses in older subjects was significantly smaller than the effect obtained in Experiment One (9 versus 19% increase, *F*(1, 12) = 6.84, *p* = 0.02). Hence, Experiment Two, like Experiment One, found evidence for an age-related reduction in the influence of sound on the bouncing-streaming percept.

## Experiment Three: Sound quality control

Experiments One and Two showed that inserting a sound into the *Ambiguous* stimulus increased Pr(“Bouncing”) responses for both younger and older subjects, but had significantly reduced impact for the older subjects. We speculated that this reduced impact might have resulted from presbycusis, age-related hearing loss. Presbycusis diminishes overall sensitivity to sound, and is characterized by a particular loss in sensitivity to high frequencies (Morrell et al., 1996). As a result, presbycusis would effectively filter out higher frequency components in the spectrum of the sharp-onset, “click” sound used in Experiments One and Two. As the perceptually sharp onset of the click depends upon the higher frequencies in its spectrum, loss of higher frequencies would make the click qualitatively less sharp. Previously, with young subjects, Grassi and Casco (2009) found that the onset attack of a sound can modify the sound’s impact on the ambiguous bouncing-streaming display. Because Grassi and Casco’s (2009) sound differed substantially from our synthesized click, we thought a test of presbycusis’ effect on our synthesized click’s ability to increase Pr(“Bouncing”) was in order. To do this test, we modified our click sound by passing it through a filter that mimicked the audiogram of an older person, and then tested a new group of younger subjects with this filtered sound.

### Methods

#### Stimuli

To modify the spectrum of the preceding experiments’ click sound, we used Matlab’s Signal Processing Toolbox (Mathworks, 2012) to construct a linear-phase, finite impulse response filter, using least squares to fit the target audiogram. We generated a filter whose pass characteristics mimicked the audiogram of the average otologically normal^2^ 70-year-old male. This average audiogram was calculated from values specified in International Organization for Standardization (ISO) 7029:2000E (prepared by Technical Committee ISO/TC 43, Acoustics). To take one example, relative to an otologically normal 18-year-old male, at 4 KHz, the audiogram of the average otologically normal 70-year-old male is −43.3 dB.

Zhou et al. (2007) showed that once the amplitude of a click was sufficient to be audible, further increases in amplitude had little or no effect on the sound’s ability to bias the percept produced by *Ambiguous* motion. But, to be safe, our test conditions included one in which the filtered sound’s amplitude had been increased to match the loudness produced by the original, unfiltered sound. To determine by how much the filtered sound had to be amplified so that its loudness matched the loudness of the original, unfiltered sound, eight additional young subjects (mean age: 18.5 years) took part in a loudness matching experiment. None of these had participated in any bouncing-streaming experiments. On each trial, subjects were presented with the original click sound (at 90 dBC) and the filtered sound with varying loudness in random order. In a two-interval forced-choice procedure, subjects identified the interval, first or second, that contained the louder sound. On every trial, the amplitude of the filtered sound was controlled by three interleaved staircases: a 1-down/1-up staircase, a 2-down/1-up staircase, and a 2-up/1-down staircase. Each staircase terminated after 20 trials, or upon reaching 12 reversals, whichever came first. The proportion of “Louder” responses was plotted as a function of dB adjustment of the filtered sound, and a Weibull function was fit to each subjects’ data to estimate the point of subjective equality (PSE), or the dB adjustment necessary to yield “Louder” responses 50% of the time. The obtained PSEs for the eight subjects ranged from 11.7 to 12.6, with a mean of 12.13 dB. That mean amplification value was used for one of the conditions in the experiment proper. Figure 1B shows the spectrogram for this amplified, filtered click.

#### Subjects

Twenty-two younger subjects were recruited for this experiment, and were compensated for their time at a rate of $10/h. None had taken part in either of the preceding experiments. As we did in those earlier experiments, we excluded subjects whose responses showed less than the expected accuracy with the unambiguous stimulus. Data from nine subjects were excluded from analyses because their accuracy in the unambiguous *Bounce* condition was <70% (accuracy range: 3–68%, average: 38%). Table 1 presents demographic information for the 13 remaining subjects.

#### Apparatus

The apparatus was the same as in Experiment One.

#### Design and procedure

There were six conditions in this Experiment: two unambiguous motion conditions (*Bounce*, *Stream*), the *Ambiguous* condition presented with no sound, and the *Ambiguous* condition accompanied by three different sounds – the original click sound from Experiment One (*Ambiguous* + *original click*), that click sound filtered to attenuate primarily high frequencies (*Ambiguous* + *filtered click*), and the filtered sound amplified by 12.13 dB to match the loudness of the unfiltered sound (*Ambiguous* + *filtered, amplified* *click*).

The procedure was the same as in Experiment One. Subjects were tested in nine blocks of trials comprising four trials of each condition in random order. Experimental trials were preceded by a practice block comprising two trials of each condition presented in random order.

## Results

**Figure 7:**
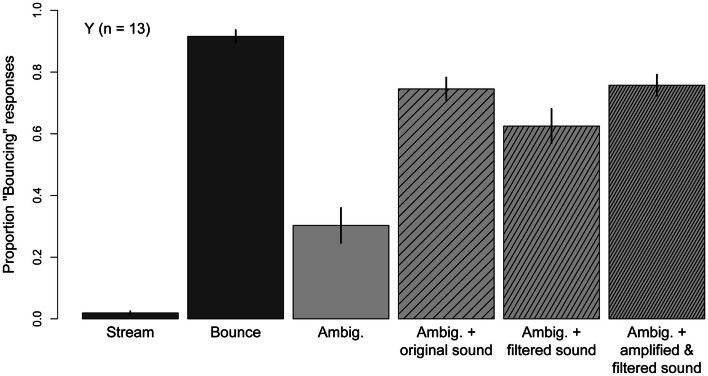
**Mean Pr(“Bouncing”) responses produced in each condition of Experiment Three: *stream* and *Bounce*; *Ambiguous*; *Ambiguous* accompanied by the sound used in Experiments One and Two; *Ambiguous* accompanied by a filtered version of the sound used in Experiments One and Two; *Ambiguous* accompanied by a filtered-and-amplified version of the sound used in Experiments One and Two**. Data are for 13 younger subjects. Error bars are ±1 standard error of the mean.

Figure 7 shows the mean proportion “Bouncing” obtained in the six conditions used in this Experiment. The two unambiguous motion conditions were successful in promoting perceptual consistency, as subjects gave 92% “Bouncing” responses in the *Bounce* condition and 98% “Streaming” responses in the *Stream* condition. Mean accuracy was significantly higher in the *Stream* condition than in the *Bounce* condition (*t*(12) = −3.03, *p* = 0.01), indicating that subjects showed a bias for “Streaming” responses. As in Experiments One and Two, the *Ambiguous* condition promoted the streaming percept (*M* = 30%) and the presentation of the original sound click biased the percept toward bouncing (*M* = 74%). A 3 (Experiment) ×  2 (sound) mixed-model ANOVA comparing performance in the *Ambiguous* and *Ambiguous* + *original Sound* conditions in Experiments One, Two, and Three revealed a significant main effect of sound (*F*(1, 30) = 96.9, *p* < 0.001), no main effect of Experiment (*F*(2, 30) = 0.04, *p* = 0.96) and no significant Experiment × Sound interaction (*F*(2, 30) = 0.47, *p* = 0.63). Thus, the proportion “Bouncing” associated with the Ambiguous condition, and the bouncing bias associated with the original sound click, show very good replicability across three separate groups of younger subjects.

Both the filtered sound (*M* = 63%) and the filtered-and-amplified sound (*M* = 76%) significantly biased the disks’ motion percept toward bouncing (*Ambiguous* + *filtered sound* versus *Ambiguous*, *t*(12) = −5.95, *p* < 0.001; *Ambiguous* + *amplified, filtered sound* versus *Ambiguous*: *t*(12) = −8.06, *p* < 0.001). The bouncing bias associated with the filtered sound was lower than that induced by the original sound (*t*(12) = 2.39, *p* = 0.03); however, the effect of the filtered-and-amplified sound was not different from the effect of the original sound (*t*(12) = −0.34, *p* = 0.74). Thus, the original click and an equivalently loud filtered click were equally effective at biasing younger subjects’ percept of the ambiguous motion stimulus toward bouncing, suggesting that age-differences in audition cannot explain the attenuated effect of the sound click observed in Experiments One and Two.

## Discussion and Conclusion

The current experiments examined age-related changes in inter- and intra-modal integration by measuring the effects of visual and auditory events on the bistable bouncing/streaming percept of a visual motion stimulus.

### Inter-modal integration

**Figure 8:**
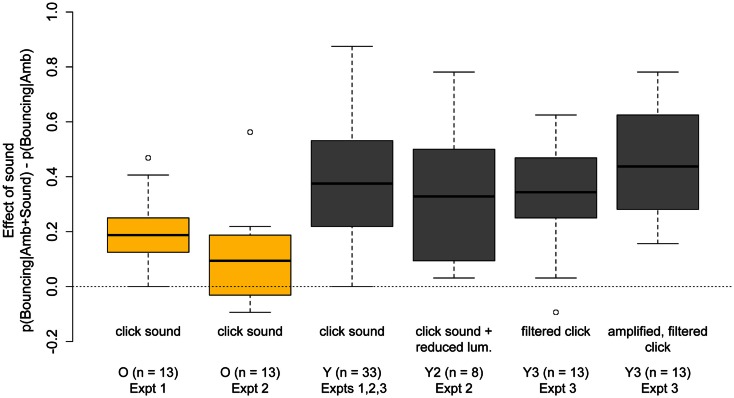
**Boxplots of the increase in Pr(“Bouncing”) responses induced by the presentation of a sound at the point of disks’ coincidence**. First and second boxplots show the bouncing bias induced by the click sound for 13 older subjects tested in Experiments One and Two. The third boxplot shows the bouncing bias induced by the click sound for 33 younger subjects across Experiments One, Two, and Three. The fourth boxplot shows the bouncing bias induced by the click sound in Experiment Two when display luminance was reduced with neutral density filters. The fifth and sixth boxplots show the bouncing bias induced by the filtered and filtered-and-amplified clicks for 13 younger subjects in Experiment Three.

Consistent with previous studies (Sekuler et al., 1997; Shimojo et al., 2001; Watanabe and Shimojo, 2001; Remijn et al., 2004; Sanabria et al., 2004; Zhou et al., 2007), presenting a brief sound at the time of disks’ coincidence strongly biased the motion percept toward bouncing in both groups. Importantly, as can be seen in Figure 8, the sound-induced bias was significantly weaker in older subjects, both in Experiments One and Two. The age-related reduction in the effect of sound on the bouncing/streaming percept is surprising given previous findings that multisensory integration is preserved, or even enhanced, in older age (Peiffer et al., 2007; Diederich et al., 2008; Mahoney et al., 2011; Winneke and Phillips, 2011, for review, see Mozolic et al., 2012), as well as the reduced ability of older subjects to inhibit task-irrelevant information (Andrés et al., 2006; Gazzaley et al., 2008).

Results in Experiments Two and Three showed that the observed age-differences cannot be explained by age-related reductions in retinal illuminance, nor by age-related hearing loss. As can be seen in Figure 8, the effect of the sound in younger subjects was highly replicable and robust to reductions in display luminance (Experiment Two boxplot) and to changes in the amplitude and frequency spectrum of the sound (Experiment Three boxplots), that were designed to mimic the effect of presbycusis in older adults.

Previous studies also have shown that the perceived timing of the sound relative to the time of disks’ coincidence is important: the sound biases the percept toward bouncing only if it occurs between 150 ms before and up to 50 ms after the disks’ coincidence (Sekuler et al., 1997; Shimojo et al., 2001). However, it is not likely that age-differences in the strength of the sound-induced bouncing bias resulted from group differences in the perceived timing of the click and visual events, as aging does not appear to affect the point of subjective simultaneity for visual and auditory stimuli (Fiacconi et al., 2013). Moreover, some studies suggest that older subjects have a wider, not narrower, time window of audio-visual integration than younger subjects (Laurienti et al., 2006; Diederich et al., 2008; Setti et al., 2011), implying that strict simultaneity of sound and visual collision would be less critical for older subjects. Thus, age-differences in perceived timing of events, if such exist, are likely to have been inconsequential for our results.

### Intra-modal integration

Experiment One also examined age-related changes in intra-modal integration in the microgenesis of the perceptual influence of a visual occluder on the bouncing/streaming percept. The sustained occluder promoted streaming in both groups, consistent with previous results (Sekuler and Sekuler, 1999; Grove and Sakurai, 2009) and with the fact that occlusion can promote perceptual continuity (Assad and Maunsell, 1995; Feldman and Tremoulet, 2006). In contrast, the brief and medium duration occluders promoted bouncing in younger subjects, with the briefest occluder inducing a stronger bouncing bias than the medium duration occluder. Interestingly, the brief and medium duration occluders did not promote bouncing in older subjects. Thus, the weakened inter-modal effect in aging was also paralleled by a reduced intra-modal effect of the transient visual occluders.

The bias toward bouncing induced by the transient visual occluder in younger subjects is consistent with several previous studies that found that transient *visual* events occurring in close spatiotemporal proximity to the point of coincidence promote bouncing (Sekuler et al., 1997; Watanabe and Shimojo, 1998; Zhou et al., 2007). Remijn and Ito (2007) showed that an increased in bouncing percepts can also be induced by occluders that do not obscure the disks’ coincidence, but that are situated in close proximity to the point of coincidence and suggested that processing the moving objects behind occluders interferes with processing of the continuity of the disks’ motion, thereby reducing the probability of a streaming percept. Kawachi et al. (2011) showed that determining whether two colored disks that intersected behind an occluder bounced off each other or streamed past each other required ≈0.2 s of post-coincidence object motion, independently of the disks’ speed, suggesting that some amount of time is necessary to match the disks’ motion across the point of coincidence. Thus, the transient occluders in Experiment One may have interfered with processing of the objects’ motion at their coincidence, resulting in an increase in bouncing percepts.

### Multiple cue combination

Finally, Experiment One also briefly examined the influence of combined auditory and visual cues on the resulting percept by concurrently presenting the medium occluder and sound click. For younger subjects, the bouncing bias induced by the combination of medium occluder and sound was equal to the bias induced by the sound alone, indicating a sub-additive effect of sound and occluder. On the other hand, the bouncing bias induced by the sound in older subjects was significantly attenuated by the presentation of the occluder, consistent with the slight reduction in proportion bouncing by the occluder alone. Thus, for both groups, performance in the combined occluder and sound condition was approximately consistent with an additive effect of the two separate cues. Zhou et al. (2007) showed that when several visual and/or auditory cues are presented with the bouncing/streaming display, the resulting percept was well predicted by a weighted sum of the effects of the cues presented in isolation. Although cue weighting varied across subjects, visual cues generally dominated auditory cues. Kawachi and Gyoba (2006) showed that intra-modal perceptual grouping of the moving disks also can override the effect of sound on the motion percept. Current results show that older subjects do combine the effects of visual and auditory cues in motion perception; however, our experiments were not designed to determine the relative weighting of these cues. Future studies should investigate whether aging affects the relative weighting of auditory and visual cues in multisensory cue combination.

### Common causes for reduced intra- and inter-modal integration

In both the intra- and inter-modal effects, integration of cues is critical, so it is possible that age-related differences in integration time might play a role. Working with younger observers, Bodelón et al. (2007) analyzed the time taken to integrate simple visual features such as color and orientation into a perceptual whole and showed that the time required to process a combination of features is longer than any individual component. If similar time constants hold for multisensory features, older subjects’ weaker integration may be explained by prolonged time required to process a combination of features. For example, older subjects require longer stimulus durations to perceive contours composed of discrete oriented elements that are embedded among distractors, suggesting that older adults require more time to integrate basic features spatially (Roudaia et al., 2011, submitted). Moreover, several studies have found changes in integration of motion signals with aging (Andersen and Ni, 2008; Roudaia et al., 2010; Arena et al., 2012). Perception of continuous object motion behind an occluder is thought to rely on the temporal integration of signals from local motion detectors tuned to the direction of motion (Bertenthal et al., 1993). Age-related changes in integration of motion signals may contribute to the reduced effect of transient occlusion on the bouncing/streaming percept in older subjects. It is interesting to note that older adults also show weaker representational momentum for motion (Piotrowski and Jakobson, 2011), a phenomenon that may be related to the effects observed here. More broadly, age-related differences in internal noise and calculation efficiency of motion detectors may affect both intra- and inter-modal effects (Bennett et al., 1999, 2007; Betts et al., 2007; Casco et al., 2012).

Some authors have suggested that transient events presented in close spatiotemporal proximity of the point of coincidence promote bouncing by disrupting the sustained attention to the disks’ motion that is necessary for the streaming percept (Watanabe and Shimojo, 1998; Shimojo et al., 2001; Kawabe and Miura, 2006). However, other studies found that removal of attention alone can not account for the effect, as some concurrent events presented at disks’ coincidence did not increase the proportion of bouncing percepts, while still distracting attention from the moving disks (Sekuler et al., 1997; Watanabe and Shimojo, 2001; Grassi and Casco, 2009). In addition, Dufour et al. (2008) showed that the presentation of a subliminal sound, an event that was unlikely to have disrupted sustained attention, also promoted the bouncing percept. Therefore, it is unlikely that differences in sustained attention can fully explain the weakened effects of the visual and auditory events.

### Reduced multisensory integration?

The most likely cause of the increase in bouncing percepts induced by the click is the integration of the sound with the visual motion stimulus into a single multisensory event (e.g., Ecker and Heller, 2005). Bushara et al. (2003) examined the neural correlates underlying the effect of sound using fMRI by comparing cortical activation for trials on which subjects reported a bouncing percept versus trials where subjects reported a streaming percept. Trials on which bouncing was perceived were accompanied by increased activation in several subcortical structures, as well as frontal and prefrontal areas, and in left posterior parietal cortex, all of which are known to be involved in multisensory processing. Conversely, trials on which streaming was perceived showed greater activation in the superior temporal gyri and occipital cortices, known to primarily process unisensory auditory and visual information, respectively. The authors suggested that the bistability of the percept arises from a competitive interaction between multisensory and unisensory areas. Consistent with these findings, Maniglia et al. (2012) showed that disrupting activity in the right posterior parietal cortex with transcranial magnetic stimulation reduced the strength of the bouncing bias that was induced by the sound, but did not affect the proportion of bouncing percepts in the silent, control condition. The authors interpreted these results as evidence for the key role of the posterior parietal cortex in the inter-modal binding of the coincidence event.

Although attentional effects alone cannot explain the effects of auditory and visual cues on the perception of the bouncing/streaming display, top-down attention has been shown to play an important role in resolving the competitive interactions between alternative percepts produced by perceptually malleable stimuli (Senkowski et al., 2005; Talsma et al., 2007, for review, see Talsma et al., 2010). Evidence for the interplay of attention and multisensory integration in the bouncing/streaming percept comes from an MEG study by Zvyagintsev et al. (2011). Similar to the study by Bushara et al. (2003), the authors presented the bouncing/streaming stimulus with a sound at the objects’ coincidence and compared activation for trials yielding each of the alternative percepts. Trials that generated a bouncing percept showed greater activity in frontal areas within 80 ms after the disks’ coincidence, followed by greater activity in the cuneus and the superior parietal lobule. Trials that generated a streaming percept showed greater activity in the auditory cortex starting 80 ms after the disks’ coincidence, closely followed by increased activity in the visual cortices and later followed by activation in the frontal areas. The authors interpreted these results as indicating that early supramodal attention mediates multisensory binding of the sound and visual stimulus to generate the bouncing percept. Thus, on trials where attention is low, multisensory binding does not occur, and the visual and auditory stimuli are processed as separate items, which yields a streaming percept.

Thus, the age-related changes in inter-modal integration shown in Figure 4 may reflect age-related declines in multisensory integration, or changes in the interaction between attentional and multisensory integration processes. Contrary to this suggestion, most studies of multisensory integration and aging show greater, not lesser multisensory enhancement in older subjects (for review, see Mozolic et al., 2012). For example, a recent MEG study (Diaconescu et al., 2013) found increased activity to audio-visual stimuli in the posterior parietal and medial prefrontal areas in older subjects, which was also correlated with the behavioral response time enhancement to audio-visual stimuli. This study highlighted the role of posterior parietal and prefrontal regions in mediating multisensory integration in older age. It is noteworthy that these same regions have been shown to be involved in the bouncing percept (Bushara et al., 2003; Zvyagintsev et al., 2011; Maniglia et al., 2012). What can account for these differential findings? Differences among measures of age-related variation in multisensory integration may be related to differences in the studies’ stimuli and tasks. Previous studies showing multisensory enhancement in older subjects primarily compared response times for detecting or discriminating brief, static unimodal or inter-modal stimuli (e.g., Laurienti et al., 2006; Peiffer et al., 2007; Mahoney et al., 2011), or examined the integration of audio-visual speech signals (e.g., Cienkowski and Carney, 2002; Maguinness et al., 2011; Winneke and Phillips, 2011). In one notable exception, Stephen et al. (2010) found evidence of reduced multisensory integration in older subjects. Interestingly, similar to our study, Stephen et al. (2010) examined the integration of sound with a visual motion stimulus. Future research should examine the possibility that aging affects multisensory interaction for static and dynamic stimuli differently.

### Conclusion

In sum, the present experiments revealed an age-related reduction in the influence of auditory and visual cues on the way that a bistable visual motion stimulus was perceived. Control experiments ruled out the possibility that this reduced influence resulted from normal, age-related sensory changes. Instead, our findings point to age-related changes in the integration of multiple cues. Because we inhabit a world in which events are defined by relationships among multiple stimuli, including stimuli from multiple senses, inter- and intra-modal integration is crucial for effective cognitive function and for successful navigation of the environment. As a result, age-related weakening of multisensory and intrasensory integration could significantly impact older adults’ performance in various aspects of everyday perception, cognition, and mobility. This suggests the importance of an expanded examination of age-related changes in cue integration more generally, both within a single sensory modality and between multiple senses. Finally, normal age-related change in vision or audition affords a potentially valuable arena within which to test theoretical accounts of the way in which multisensory integration tracks changes in the reliability of information provided by one sense or another (Ernst and Banks, 2002; Gori et al., 2012).

## Conflict of Interest Statement

The authors declare that the research was conducted in the absence of any commercial or financial relationships that could be construed as a potential conflict of interest.

## Footnotes

^1^The sound is more properly categorized as a *pluck*, but for consistency with previous reports we opt to use the term *click*.

^2^ISO 7029-2000 defines otologically normal as “a person in a normal state of health who is free from all signs or symptoms of ear disease and from obstructing wax in the ear canals, and who has no history of undue exposure to noise.”
